# Supplementary material for: ABCF ATPases Involved in Protein Synthesis, Ribosome Assembly and Antibiotic Resistance: Structural and Functional Diversification across the Tree of Life
Source: J Mol Biol. 2019 Aug 23;431(18):3568–90. doi: 10.1016/j.jmb.2018.12.013 (PMC6723617; doi:10.1016/j.jmb.2018.12.013)
Supplement: Supplementary file 1 — S1 Table. Taxonomy of 4505 species, and their ABCF composition. All species considered in the analysis are listed, ordered by taxonomy. The number and identity of ABCF subfamilies are recorded. S2 Table. Classification of 16,848 ABCF sequences into subfamilies, accompanied by domain assignments. The unique sequence identifiers are included for retrieval of data from online repositories. S3 Table. Domain coordinates. The domain coordinates for the representative sequences in Fig. 3A are listed. In the second tab, all the coordinates for each identified domain in all ABCFs are given. As these are from HMM hits, the same domain can have more than one hit in each protein. For example, the ABC1 HMM always hits the ABC2 domain, and vice versa. Duplicate domain hits were removed when generating Fig. 3A. S4 Table. Presence and absence of EFL, eEF1A and eEF3 in eukaryotes. Where the distribution is unchanged within a specific taxonomic lineage, those rows are collapsed down to one, and the highest common taxonomic rank is given. The full lineage data are available in the second tab. S5 Table. Transit peptide predictions. Predictions were made separately for plastid-containing and non-plastid containing eukaryotes. The description of the output format is shown below the predictions. Table S6. Primers used in the study Table S7. The starting OD600 of E. coli CFT073 and its derivatives S1 Figure. Ladderized version of the Fig. 1 tree. All branch support values and taxon names including subfamily identity are shown. Branch coloring is as per Fig. 1. Orange stars show Uup sequences that have truncated Arm subdomains. S2 Figure. Sequence logos of domains show amino acid biases. Sequence logos of each domain HMM. The height of stacked amino acids at each position show the information content, in bits, with letters dividing the height according to their estimated probability. Beneath the stacked amino acids there are three lines showing probabilities; line 1 is occupancy, the probab [file mmc1.zip › SI 2/FigS6.pdf]

Figure S6

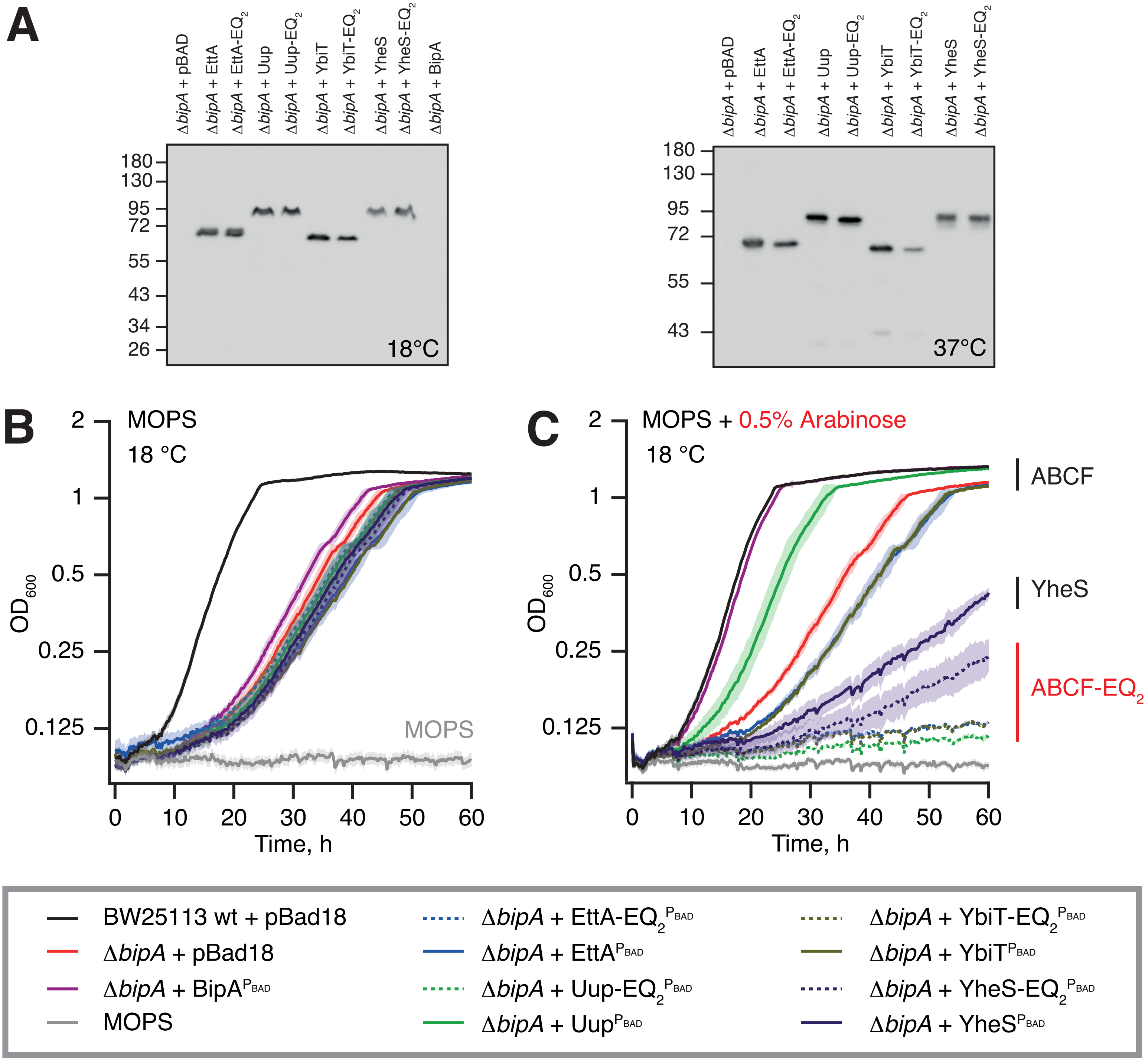
A
ΔbipA + pBAD
ΔbipA + EttA
ΔbipA + EttA-EQ2
ΔbipA + Uup
ΔbipA + Uup-EQ2
ΔbipA + YbiT
ΔbipA + YbiT-EQ2
ΔbipA + YheS
ΔbipA + YheS-EQ2
ΔbipA + BipA
180
130
95
72
55
43
34
26
18°C
180
130
95
72
55
43
37°C
B
MOPS
18 °C
OD600
Time, h
MOPS
C
MOPS + 0.5% Arabinose
18 °C
ABCF
YheS
ABCF-EQ2
BW25113 wt + pBad18
ΔbipA + pBad18
ΔbipA + BipA PBAD
MOPS
ΔbipA + EttA-EQ2 PBAD
ΔbipA + EttA PBAD
ΔbipA + Uup-EQ2 PBAD
ΔbipA + Uup PBAD
ΔbipA + YbiT-EQ2 PBAD
ΔbipA + YbiT PBAD
ΔbipA + YheS-EQ2 PBAD
ΔbipA + YheS PBAD
