# Supplementary material for: ABCF ATPases Involved in Protein Synthesis, Ribosome Assembly and Antibiotic Resistance: Structural and Functional Diversification across the Tree of Life
Source: J Mol Biol. 2019 Aug 23;431(18):3568–90. doi: 10.1016/j.jmb.2018.12.013 (PMC6723617; doi:10.1016/j.jmb.2018.12.013)
Supplement: Supplementary file 1 — S1 Table. Taxonomy of 4505 species, and their ABCF composition. All species considered in the analysis are listed, ordered by taxonomy. The number and identity of ABCF subfamilies are recorded. S2 Table. Classification of 16,848 ABCF sequences into subfamilies, accompanied by domain assignments. The unique sequence identifiers are included for retrieval of data from online repositories. S3 Table. Domain coordinates. The domain coordinates for the representative sequences in Fig. 3A are listed. In the second tab, all the coordinates for each identified domain in all ABCFs are given. As these are from HMM hits, the same domain can have more than one hit in each protein. For example, the ABC1 HMM always hits the ABC2 domain, and vice versa. Duplicate domain hits were removed when generating Fig. 3A. S4 Table. Presence and absence of EFL, eEF1A and eEF3 in eukaryotes. Where the distribution is unchanged within a specific taxonomic lineage, those rows are collapsed down to one, and the highest common taxonomic rank is given. The full lineage data are available in the second tab. S5 Table. Transit peptide predictions. Predictions were made separately for plastid-containing and non-plastid containing eukaryotes. The description of the output format is shown below the predictions. Table S6. Primers used in the study Table S7. The starting OD600 of E. coli CFT073 and its derivatives S1 Figure. Ladderized version of the Fig. 1 tree. All branch support values and taxon names including subfamily identity are shown. Branch coloring is as per Fig. 1. Orange stars show Uup sequences that have truncated Arm subdomains. S2 Figure. Sequence logos of domains show amino acid biases. Sequence logos of each domain HMM. The height of stacked amino acids at each position show the information content, in bits, with letters dividing the height according to their estimated probability. Beneath the stacked amino acids there are three lines showing probabilities; line 1 is occupancy, the probab [file mmc1.zip › SI 2/S1_Materials_and_Methods_180718.docx]

**S1 Supplementary Methods**

**ABCF ATPases involved in protein synthesis, ribosome assembly and antibiotic resistance: structural and functional diversification across the tree of life**

Victoriia Murina*^1,2^, Marje Kasari*^1^, Hiraku Takada^1,2^, Mariliis Hinnu^3^, Chayan Kumar Saha^1^, James W. Grimshaw^4^, Takahiro Seki^5^, Michael Reith^1^, Marta Putrinš^3^, Tanel Tenson^3^, Henrik Strahl^4^, Vasili Hauryliuk^1,2,3^ and Gemma Catherine Atkinson^†1^

^1^ Department of Molecular Biology, Umeå University, 901 87, Umeå, Sweden

^2^ Laboratory for Molecular Infection Medicine Sweden (MIMS), Umeå University, 901 87, Umeå, Sweden

^3^ University of Tartu, Institute of Technology, Nooruse 1, 50411 Tartu, Estonia

^4^ Centre for Bacterial Cell Biology, Institute for Cell and Molecular Biosciences Newcastle University, Richardson Road, Newcastle upon Tyne, NE2 4AX, United Kingdom

^5^ Department of Applied Chemistry and Biotechnology, Faculty of Engineering, Chiba University, 263-8522, Chiba, Japan

**Construction of plasmids and bacterial strains**

All bacterial strains and plasmids used in this study are listed in **Table 2**.

Construction of pBAD-based expression plasmids: Cloning of the pBAD-FTH-EttA_EQ2_ plasmid was ordered from GeneArt AG (LifeTechnologies). The nucleotide sequence encoding 3xFLAG-TEV-His_6_ tag (FTH: 5’-ATGGACTACAAAGACCATGACGGTGATTATAAAGATCATGACATCGATTACAAGGATGACGATGACAAAGGCGGTGAAAACCTGTATTTTCAGGGTGTACATCATCATCATCATCACGGCGCCGGC-3’) was fused to the 5’ end of the *ettA* (*yjjK*) ORF instead of the native start codon using synthetic oligonucleotides and/or PCR products. The two point mutations E188Q and E470Q were introduced to the ATP-binding sites of EttA to yield the EttA_EQ2_ mutant. The fragment was inserted into pBADMyc_HisB_A264 vector (Invitrogen) using restriction sites NcoI and XbaI. In the resulting construct, the C-terminal Myc_HisB tag is not expressed due to the introduction of a stop codon. ORFs encoding *ybiT*, *yheS* and *uup* were PCR amplified from the corresponding pCAN24-based expression constructs (ASKA collection [1]) and sub-cloned into pBAD-FTH vectors using KasI and XbaI restriction sites (cloning was done in Protein Expertise Platform (PEP), Umeå University). EQ_2_ mutations were introduced using site-directed mutagenesis (E182Q and E466Q in Uup, E182Q and E465Q in YbiT, E175Q and E456Q in YheS), EttA wild type was obtained by reverse mutagenesis from the pBAD-FTH-EttA_EQ2_ plasmid. All cloning was performed by PEP, Umeå University. The Uup expression construct obtained from the ASKA collection contained A145G and A236G mutations, which were reverted to wild type by site-directed mutagenesis.

Construction of pSC101-based expression plasmids: All cloning was performed using CPEC method [2]. First, the PybaJ promoter driving expression of Timer fluorescent protein [3] in pSC101-based [4, 5] low-copy pSC101-PybaJ-Timer plasmid [6] was replaced with constitutive tet-promoter (Ptet) [7] originating from the pBR322 plasmid [8], yielding pSC101-Ptet-Timer. Second, the Timer ORF in pSC101-Ptet-Timer plasmid was replaced with ABCF genes of interest – *ettA*, *uup*, *ybiT* and *yheS* – amplified together with their native Shine-Dalgarno elements from *E. coli* CFT073 [9] genomic DNA (accession number AE014075). Amplification primers were designed to change the initiation codons of *bipA*, *ettA* and *ybiT* genes to ATG. Primer sequences are listed in **Table S6**. The full nucleotide sequence of the pSC101-Ptet-Timer plasmid is provided below.

ABCF gene knockouts and overexpression constructs in CFT073: Targeted gene knockouts were generated in the uropathogenic *E. coli* strain CFT073 [9] using the λRed-mediated gene disruption method [10]. Briefly, a kanamycin resistance cassette was PCR amplified from pKD4 or pKD13 with 50-base pair overhangs specific to the start and end of each targeted locus. PCR products were introduced via electroporation into CFT073 carrying the pKD46 plasmid which encodes an arabinose-inducible λRed recombinase. From all constructed strains the kanamycin resistance cassette was removed by using the FLP recombinase expression plasmid pCP20. Gene knockouts were confirmed by PCR. Primer sequences are listed in **Table S6**.

Construction of *vmlR* marker-less deletion mutant: Strain VHB5 [*trpC2* Δ*vmlR*] was constructed using the marker-free deletion technique [11] in wild type *B. subtilis* 168 background [12]. First, three linear ≈500 nt-long DNA fragments were amplified by PCR using genomic DNA as a template: one located upstream of the *vmlR* ORF (primers VmlR-A-F and VmlR-A-R), one downstream of the *vmlR* ORF (primers VmlR-B-F and VmlR-B-R) and one within the *vmlR* ORF (primers VmlR-C-F and VmlR-C-R). Second, the TMO310 *mazF* cassette was amplified by PCR using primers chpA-R and pAPNC-F. The cassette contains i) the *mazF* toxin ORF under the control of an IPTG-inducible promoter (P*_spac_*), ii) the *lacI* ORF for expression of Lac repressor controlling the P*_spac_,* and iii) the spectinomycin resistance marker (*spc^r^*). All four PCR products described above were used simultaneously as the template for PCR amplification using primers VmlR-A-F and VmlR-C-R. The resultant long PCR fragment was used to transform the *B. subtilis* strain 168. *vmlR* deletion mutants were selected by spectinomycin resistance, followed by a second selection step on IPTG plates to identify marker-less *vmlR* deletion mutants lacking the *mazF* toxin ORF, yielding the VHB5 strain. Primer sequences are listed in **Table S6**.

Construction of IPTG-inducible *vmlR-HTF* and *vmlREQ_2_-HTF* strains in *B. subtilis* Δ*vmlR* background: To construct VHB91 [*trpC2* Δ*vmlR* *thrC*::P*_hy-spank_*-*vmlR-HTF Kan^r^*] and VHB92 [*trpC2* Δ*vmlR* *thrC*::P*_hy-spank_*-*vmlREQ_2_-HTF Kan^r^*] strains expressing C-terminally His_6_-TEV-3xFLAG-tagged [HTF: 5’- GGCGGCCATCATCATCATCATCACGCCAAAGGCGGTGAAAACCTGTATTTTCAGGGTGTAGCCGACTACAAAGACCATGACGGTGATTATAAAGATCATGACATCGATTACAAGGATGACGATGACAAAGGC-3’] wild type or EQ_2_ mutant VmlR under the control of an IPTG-inducible P*_hy-spank_* promotor [13], DNA fragments encoding either *vmlR-HTF* or *vmlREQ_2_-HTF* were PCR-amplified from VHp62 (pAPNC-*vmlR-HTF*) or VHp66 (pAPNC-*vmlREQ_2_-HTF*) plasmid, respectively. To generate the VmlR_EQ2_ mutant, two point mutations (E129Q and E432Q) were introduced to the ATP-binding sites of VmlR. The two fragments were inserted into pHT009 plasmid encoding a kanamycin-resistance marker, a polylinker downstream of the P*_hy-spank_* promoter and the *lac* repressor ORF – all inserted in the middle of the *thrC* gene – using restriction sites HindIII and SphI. The resultant plasmids pHT009-*vmlR-HTF* and pHT009-*vmlREQ_2_-HTF* were used to transform the VHB5 [*trpC2* Δ*vmlR*] strain. Selection for kanamycin resistance yielded the desired VHB91 and VHB92 strains, respectively. pHT009 is *amyE*-integration plasmid encoding an IPTG-inducible P*_hy-spank_* promotor. The plasmid was constructed by PCR-linearizing the plasmid pHT003 [14] with Primer VHT_17 and VHT_18, by amplifying P_rrnO_-Kan^r^ coding sequence from chromosomal DNA of *B. subtilis* RIK1069 [15] with Primer VHT_15 and VHT_16, and by fusing the products together with the help of NEBuilder HiFi DNA Assembly master mix (New England BioLabs, Ipswich, MA) yielding pHT009. Primer sequences are listed in **Supplementary Table 2**.

Construction of *B. subtilis* for IPTG-inducible expression of untagged *vmlR* and *vmlREQ_2_* in Δ*vmlR* mutant background: To construct VHB44 [*trpC2* Δ*vmlR* *thrC*::P*_hy-spank_*-*vmlR* Kan^r^] and VHB45 [*trpC2* Δ*vmlR* *thrC*::P*_hy-spank_*-*vmlREQ_2_* Kan^r^] strains, untagged VmlR under the control of an IPTG-inducible P*_hy-spank_* promotor, a PCR product encoding *vmlREQ2* was PCR-amplified from the VHp63 plasmid (pAPNC-*vmlREQ*2-HTF) VHp62 (pAPNC-*vmlR-HTF*) or VHp66 (pAPNC-*vmlREQ_2_-HTF*) plasmid, respectively, using the primers PhyvmlR_F and PhyvmlR_R. The second PCR fragment encoding a kanamycin-resistance marker, a polylinker downstream of the P*_hy-spank_* promoter and the lac repressor ORF – all inserted in the middle of the *thrC* gene – was PCR-amplified from pHT009 plasmid using primers pHT002_F and pHT002_R. The two fragments were ligated using the NEBuilder HiFi DNA Assembly master mix (New England BioLabs, Ipswich, MA) yielding pHT009-*vmlRE* and pHT009-*vmlREQ2* plasmids were used to transform the VHB5 [*trpC2* Δ*vmlR*] strain. Selection for kanamycin resistance yielded desired strains VHB44 and VHB45. Primer sequences are listed in **Table S6**.

Construction of xylose-inducible *vmlR*-NeoGreen strain in *B. subtilis* Δ*vmlR* mutant background: To construct the VHB38 [*trpC2* Δ*vmlR* *amyE*::P*_xyl_*-*vmlR-mNeoGreen Spc^r^*] *vmlR* ORF was amplified from *B. subtilis* chromosomal DNA. The fragment was inserted into pSHP2 plasmid in the middle of the *amyE* gene using restriction sites ApaI and EcoRI. The resultant plasmid psHP2-*vmlR* was used to transform the VHB5 [*trpC2* Δ*vmlR*] strain. Selection for spectinomycin resistance yielded the desired VHB38 strain. pSHP2 is an *amyE*-integration plasmid encoding a xylose inducible promoter followed by a multiple cloning site and mNeonGreen fluorescent protein [16] carrying an *in-frame* 7 amino acid flexible linker (SGSGSGS)*.* The plasmid was constructed by PCR-linearizing the plasmid pSG1154 [17] with oligos pSG1154_fwd and pSG1154_rev, by amplifying mNeonGreen coding sequence from chromosomal DNA of *B. subtilis* bAB185 encoding *mNG-ftsZ*  [18] with oligos mNG_fwd and mNG _rev, and by fusing the products together with the help of In‑Fusion HD Cloning Kit (Clontech) followed by transformation into *E. coli*.  All PCR reaction were carried out with Q5 polymerase (NEB) and the sequence of the relevant plasmid region (*Pxyl-mcs-mNG*) was verified by sequencing. All primer sequences are listed in **Table S6**.

**Nucleotide sequence of the pSC101-Ptet-Timer plasmid [6].** Timer ORF is shown in bold.

ctagaggcatcaaataaaacgaaaggctcagtcgaaagactgggcctttcgttttatctgttgtttgtcggtgaacgctctcctgagtaggacaaatccgccgccctagacctagggtacgggttttgctgcccgcaaacgggctgttctggtgttgctagtttgttatcagaatcgcagatccggcttcaggtttgccggctgaaagcgctatttcttccagaattgccatgattttttccccacgggaggcgtcactggctcccgtgttgtcggcagctttgattcgataagcagcatcgcctgtttcaggctgtctatgtgtgactgttgagctgtaacaagttgtctcaggtgttcaatttcatgttctagttgctttgttttactggtttcacctgttctattaggtgttacatgctgttcatctgttacattgtcgatctgttcatggtgaacagctttaaatgcaccaaaaactcgtaaaagctctgatgtatctatcttttttacaccgttttcatctgtgcatatggacagttttccctttgatatctaacggtgaacagttgttctacttttgtttgttagtcttgatgcttcactgatagatacaagagccataagaacctcagatccttccgtatttagccagtatgttctctagtgtggttcgttgtttttgcgtgagccatgagaacgaaccattgagatcatgcttactttgcatgtcactcaaaaattttgcctcaaaactggtgagctgaatttttgcagttaaagcatcgtgtagtgtttttcttagtccgttacgtaggtaggaatctgatgtaatggttgttggtattttgtcaccattcatttttatctggttgttctcaagttcggttacgagatccatttgtctatctagttcaacttggaaaatcaacgtatcagtcgggcggcctcgcttatcaaccaccaatttcatattgctgtaagtgtttaaatctttacttattggtttcaaaacccattggttaagccttttaaactcatggtagttattttcaagcattaacatgaacttaaattcatcaaggctaatctctatatttgccttgtgagttttcttttgtgttagttcttttaataaccactcataaatcctcatagagtatttgttttcaaaagacttaacatgttccagattatattttatgaatttttttaactggaaaagataaggcaatatctcttcactaaaaactaattctaatttttcgcttgagaacttggcatagtttgtccactggaaaatctcaaagcctttaaccaaaggattcctgatttccacagttctcgtcatcagctctctggttgctttagctaatacaccataagcattttccctactgatgttcatcatctgagcgtattggttataagtgaacgataccgtccgttctttccttgtagggttttcaatcgtggggttgagtagtgccacacagcataaaattagcttggtttcatgctccgttaagtcatagcgactaatcgctagttcatttgctttgaaaacaactaattcagacatacatctcaattggtctaggtgattttaatcactataccaattgagatgggctagtcaatgataattactagtccttttcctttgagttgtgggtatctgtaaattctgctagacctttgctggaaaacttgtaaattctgctagaccctctgtaaattccgctagacctttgtgtgttttttttgtttatattcaagtggttataatttatagaataaagaaagaataaaaaaagataaaaagaatagatcccagccctgtgtataactcactactttagtcagttccgcagtattacaaaaggatgtcgcaaacgctgtttgctcctctacaaaacagaccttaaaaccctaaaggcttaagtagcaccctcgcaagctcgggcaaatcgctgaatattccttttgtctccgaccatcaggcacctgagtcgctgtctttttcgtgacattcagttcgctgcgctcacggctctggcagtgaatgggggtaaatggcactacaggcgccttttatggattcatgcaaggaaactacccataatacaagaaaagcccgtcacgggcttctcagggcgttttatggcgggtctgctatgtggtgctatctgactttttgctgttcagcagttcctgccctctgattttccagtctgaccacttcggattatcccgtgacaggtcattcagactggctaatgcacccagtaaggcagcggtatcatcaacaggcttacccgtcttactgtccctagtgcttggattctcaccaataaaaaacgcccggcggcaaccgagcgttctgaacaaatccagatggagttctgaggtcattactggatctatcaacaggagtccaagcgagctctcgaaccccagagtcccgctcagaagaactcgtcaagaaggcgatagaaggcgatgcgctgcgaatcgggagcggcgataccgtaaagcacgaggaagcggtcagcccattcgccgccaagctcttcagcaatatcacgggtagccaacgctatgtcctgatagcggtccgccacacccagccggccacagtcgatgaatccagaaaagcggccattttccaccatgatattcggcaagcaggcatcgccatgggtcacgacgagatcctcgccgtcgggcatgcgcgccttgagcctggcgaacagttcggctggcgcgagcccctgatgctcttcgtccagatcatcctgatcgacaagaccggcttccatccgagtacgtgctcgctcgatgcgatgtttcgcttggtggtcgaatgggcaggtagccggatcaagcgtatgcagccgccgcattgcatcagccatgatggatactttctcggcaggagcaaggtgagatgacaggagatcctgccccggcacttcgcccaatagcagccagtcccttcccgcttcagtgacaacgtcgagcacagctgcgcaaggaacgcccgtcgtggccagccacgatagccgcgctgcctcgtcctgcagttcattcagggcaccggacaggtcggtcttgacaaaaagaaccgggcgcccctgcgctgacagccggaacacggcggcatcagagcagccgattgtctgttgtgcccagtcatagccgaatagcctctccacccaagcggccggagaacctgcgtgcaatccatcttgttcaatcatgcgaaacgatcctcatcctgtctcttgatcagatcttgatcccctgcgccatcagatccttggcggcaagaaagccatccagtttactttgcagggcttcccaaccttaccagagggcgccccagctggcaattccgacgtctaagaaaccattattatcatgacattaacctataaaaataggcgtatcacgaggccctttcgtcttcacctcgaagctt**ctacaggaacaggtggtagcggccctcggtgcgttcgtactgctccacgatggtgtagtcctcgttgtgggaggtgatgtccagcttggtgtccacgtagtagtagccgggcagttgcacgggcttcttggccatgtagatggacttgaactccaccaggtagtggccgccgtccttcagcttcagggccttgtggatctcgcccttcagcacgccgtcgcgggggtacaggcgctcggtggagggctcccagcccatagtcttcttctgcattacggggccgtcggaggggaagttcacgccgatgaacttcaccttgtagatgaagcagccgtcctgcagggaggagtcctgggtcacggtcaccacgccgccgtcctcgaagttcatcacgcgctcccacttgaagccctcggggaaggacagcttcttgtagtcggggatgtcggcggggtgcttcacgtacaccttggagccgtactggaactggggggacaggatgtcccaggcgaagggcagggggccgcccttggtcaccttcagcttggcggtctgggtgccctcgtaggggcggccctcgccctcgccctcgatctcgaactcgtggccgttcacggagccctccatgcgcactttgaagcgcatgaactccttgatgacgtcctcggtggatgccat**atgtatatctccttctgcgttagcaatttaactgtgataaactaccgcattaaagcttatcgatgataagctgtcaaacatgagaatctacgccggacgcatcgtggccggcatcaccggcgccacaggtgcggttgctggcgcctatatcgccgacatcaccgatggggaagatcgggctcgccacttcgggctcatgagcgcttgtttcggcgtgggtatggtggcaggccccgtggccgggggactgttgggcgccatctccttgggcatgcaagct

**Supplementary references**

1. Kitagawa, M., et al., *Complete set of ORF clones of Escherichia coli ASKA library (a complete set of E. coli K-12 ORF archive): unique resources for biological research.* DNA Res, 2005. **12**(5): p. 291-9.

2. Quan, J. and J. Tian, *Circular polymerase extension cloning for high-throughput cloning of complex and combinatorial DNA libraries.* Nat Protoc, 2011. **6**(2): p. 242-51.

3. Terskikh, A., et al., *"Fluorescent timer": protein that changes color with time.* Science, 2000. **290**(5496): p. 1585-8.

4. Lee, T.S., et al., *BglBrick vectors and datasheets: A synthetic biology platform for gene expression.* J Biol Eng, 2011. **5**: p. 12.

5. Cohen, S.N., et al., *Construction of biologically functional bacterial plasmids in vitro.* Proc Natl Acad Sci U S A, 1973. **70**(11): p. 3240-4.

6. Claudi, B., et al., *Phenotypic variation of Salmonella in host tissues delays eradication by antimicrobial chemotherapy.* Cell, 2014. **158**(4): p. 722-733.

7. Harley, C.B., et al., *Transcription initiation at the tet promoter and effect of mutations.* Nucleic Acids Res, 1988. **16**(15): p. 7269-85.

8. Balbas, P., et al., *Plasmid vector pBR322 and its special-purpose derivatives--a review.* Gene, 1986. **50**(1-3): p. 3-40.

9. Welch, R.A., et al., *Extensive mosaic structure revealed by the complete genome sequence of uropathogenic Escherichia coli.* Proc Natl Acad Sci U S A, 2002. **99**(26): p. 17020-4.

10. Datsenko, K.A. and B.L. Wanner, *One-step inactivation of chromosomal genes in Escherichia coli K-12 using PCR products.* Proc Natl Acad Sci U S A, 2000. **97**(12): p. 6640-5.

11. Morimoto, T., et al., *A simple method for introducing marker-free deletions in the Bacillus subtilis genome.* Methods Mol Biol, 2011. **765**: p. 345-58.

12. Barbe, V., et al., *From a consortium sequence to a unified sequence: the Bacillus subtilis 168 reference genome a decade later.* Microbiology, 2009. **155**(Pt 6): p. 1758-75.

13. Britton, R.A., et al., *Genome-wide analysis of the stationary-phase sigma factor (sigma-H) regulon of Bacillus subtilis.* J Bacteriol, 2002. **184**(17): p. 4881-90.

14. Takada, H., et al., *An essential enzyme for phospholipid synthesis associates with the Bacillus subtilis divisome.* Mol Microbiol, 2014. **91**(2): p. 242-55.

15. Tagami, K., et al., *Expression of a small (p)ppGpp synthetase, YwaC, in the (p)ppGpp(0) mutant of Bacillus subtilis triggers YvyD-dependent dimerization of ribosome.* Microbiologyopen, 2012. **1**(2): p. 115-34.

16. Shaner, N.C., et al., *A bright monomeric green fluorescent protein derived from Branchiostoma lanceolatum.* Nat Methods, 2013. **10**(5): p. 407-9.

17. Lewis, P.J. and A.L. Marston, *GFP vectors for controlled expression and dual labelling of protein fusions in Bacillus subtilis.* Gene, 1999. **227**(1): p. 101-10.

18. Bisson-Filho, A.W., et al., *Treadmilling by FtsZ filaments drives peptidoglycan synthesis and bacterial cell division.* Science, 2017. **355**(6326): p. 739-743.
