# Supplementary material for: ABCF ATPases Involved in Protein Synthesis, Ribosome Assembly and Antibiotic Resistance: Structural and Functional Diversification across the Tree of Life
Source: J Mol Biol. 2019 Aug 23;431(18):3568–90. doi: 10.1016/j.jmb.2018.12.013 (PMC6723617; doi:10.1016/j.jmb.2018.12.013)
Supplement: Supplementary file 1 — S1 Table. Taxonomy of 4505 species, and their ABCF composition. All species considered in the analysis are listed, ordered by taxonomy. The number and identity of ABCF subfamilies are recorded. S2 Table. Classification of 16,848 ABCF sequences into subfamilies, accompanied by domain assignments. The unique sequence identifiers are included for retrieval of data from online repositories. S3 Table. Domain coordinates. The domain coordinates for the representative sequences in Fig. 3A are listed. In the second tab, all the coordinates for each identified domain in all ABCFs are given. As these are from HMM hits, the same domain can have more than one hit in each protein. For example, the ABC1 HMM always hits the ABC2 domain, and vice versa. Duplicate domain hits were removed when generating Fig. 3A. S4 Table. Presence and absence of EFL, eEF1A and eEF3 in eukaryotes. Where the distribution is unchanged within a specific taxonomic lineage, those rows are collapsed down to one, and the highest common taxonomic rank is given. The full lineage data are available in the second tab. S5 Table. Transit peptide predictions. Predictions were made separately for plastid-containing and non-plastid containing eukaryotes. The description of the output format is shown below the predictions. Table S6. Primers used in the study Table S7. The starting OD600 of E. coli CFT073 and its derivatives S1 Figure. Ladderized version of the Fig. 1 tree. All branch support values and taxon names including subfamily identity are shown. Branch coloring is as per Fig. 1. Orange stars show Uup sequences that have truncated Arm subdomains. S2 Figure. Sequence logos of domains show amino acid biases. Sequence logos of each domain HMM. The height of stacked amino acids at each position show the information content, in bits, with letters dividing the height according to their estimated probability. Beneath the stacked amino acids there are three lines showing probabilities; line 1 is occupancy, the probab [file mmc1.zip › SI 2/TableS5_transit_peptides.docx]

**Table S3. Transit peptide predictions**

| **Name** | **Len** | **cTP** | **mTP** | **SP** | **other** | **Loc** | **RC** |
| --- | --- | --- | --- | --- | --- | --- | --- |
| mEttA_Phytophthora_c | 589 |  | 0.925 | 0.012 | 0.122 | M | 1 |
| mEttA_Phytophthora_c | 594 |  | 0.944 | 0.007 | 0.123 | M | 1 |
| mEttA_Phytophthora_i | 590 |  | 0.917 | 0.015 | 0.147 | M | 2 |
| mEttA_Dictyostelium_ | 646 |  | 0.479 | 0.031 | 0.599 | _ | 5 |
| mEttA_Dictyostelium_ | 658 |  | 0.905 | 0.015 | 0.158 | M | 2 |
| mEttA_Acanthamoeba_c | 593 |  | 0.131 | 0.036 | 0.917 | _ | 2 |
| mEttA_Emiliania_huxl | 676 |  | 0.116 | 0.077 | 0.847 | _ | 2 |
| mEttA_Saprolegnia_di | 564 |  | 0.75 | 0.053 | 0.156 | M | 3 |
| mEttA_Aphanomyces_in | 569 |  | 0.911 | 0.036 | 0.123 | M | 2 |
| mEttA_Phytophthora_p | 603 |  | 0.917 | 0.014 | 0.17 | M | 2 |
| mEttA_Phytophthora_s | 593 |  | 0.942 | 0.01 | 0.106 | M | 1 |
| mEttA_Aphanomyces_as | 586 |  | 0.846 | 0.046 | 0.193 | M | 2 |
| mEttA_Aphanomyces_as | 570 |  | 0.846 | 0.046 | 0.193 | M | 2 |
| mEttA_Dictyostelium_ | 652 |  | 0.589 | 0.025 | 0.558 | M | 5 |
| algAF1_Fragilariopsi | 553 | 0.044 | 0.291 | 0.07 | 0.768 | _ | 3 |
| algAF1_Fragilariopsi | 640 | 0.207 | 0.016 | 0.931 | 0.016 | S | 2 |
| algAF1_Fragilariopsi | 789 | 0.02 | 0.872 | 0.042 | 0.236 | M | 2 |
| algAF1_Pseudo-nitzsc | 706 | 0.032 | 0.566 | 0.044 | 0.688 | _ | 5 |
| algAF1_Pseudo-nitzsc | 597 | 0.036 | 0.283 | 0.051 | 0.65 | _ | 4 |
| algAF1_Ostreococcus_ | 582 | 0.045 | 0.062 | 0.47 | 0.675 | _ | 4 |
| algAF1_Ostreococcus_ | 512 | 0.089 | 0.106 | 0.101 | 0.883 | _ | 2 |
| algAF1_Physcomitrell | 481 | 0.077 | 0.273 | 0.035 | 0.633 | _ | 4 |
| algAF1_Phaeodactylum | 525 | 0.063 | 0.176 | 0.039 | 0.867 | _ | 2 |
| algAF1_Phaeodactylum | 518 | 0.333 | 0.115 | 0.044 | 0.124 | C | 4 |
| algAF1_Thalassiosira | 575 | 0.034 | 0.468 | 0.042 | 0.767 | _ | 4 |
| algAF1_Micromonas_sp | 692 | 0.104 | 0.123 | 0.048 | 0.813 | _ | 2 |
| algAF1_Micromonas_sp | 582 | 0.082 | 0.072 | 0.378 | 0.567 | _ | 5 |
| algAF1_Volvox_carter | 937 | 0.962 | 0.116 | 0.004 | 0.042 | C | 1 |
| algAF1_Volvox_carter | 715 | 0.951 | 0.104 | 0.006 | 0.045 | C | 1 |
| algAF1_Micromonas_pu | 574 | 0.053 | 0.118 | 0.091 | 0.903 | _ | 2 |
| algAF1_Micromonas_pu | 577 | 0.071 | 0.227 | 0.041 | 0.75 | _ | 3 |
| algAF1_Ostreococcus_ | 1048 | 0.034 | 0.108 | 0.29 | 0.846 | _ | 3 |
| algAF1_Ostreococcus_ | 442 | 0.06 | 0.221 | 0.568 | 0.133 | S | 4 |
| algAF1_Ostreococcus_ | 406 | 0.486 | 0.771 | 0.021 | 0.019 | M | 4 |
| algAF1_Emiliania_hux | 640 | 0.528 | 0.54 | 0.016 | 0.021 | M | 5 |
| algAF1_Emiliania_hux | 761 | 0.094 | 0.036 | 0.13 | 0.688 | _ | 3 |
| algAF1_Emiliania_hux | 553 | 0.07 | 0.05 | 0.146 | 0.692 | _ | 3 |
| algAF1_Guillardia_th | 478 | 0.382 | 0.198 | 0.061 | 0.013 | C | 5 |
| algAF1_Aureococcus_a | 1031 | 0.119 | 0.551 | 0.249 | 0.006 | M | 4 |
| algUup_Fragilariopsi | 680 | 0.098 | 0.48 | 0.027 | 0.368 | M | 5 |
| algUup_Fragilariopsi | 635 | 0.098 | 0.48 | 0.027 | 0.368 | M | 5 |
| algUup_Fragilariopsi | 522 | 0.052 | 0.412 | 0.033 | 0.683 | _ | 4 |
| algUup_Fragilariopsi | 541 | 0.051 | 0.376 | 0.046 | 0.713 | _ | 4 |
| algUup_Pseudo-nitzsc | 712 | 0.624 | 0.025 | 0.183 | 0.464 | C | 5 |
| algUup_Ostreococcus_ | 723 | 0.879 | 0.484 | 0.006 | 0.034 | C | 4 |
| algUup_Chlamydomonas | 626 | 0.19 | 0.039 | 0.186 | 0.716 | _ | 3 |
| algUup_Phaeodactylum | 679 | 0.662 | 0.026 | 0.11 | 0.381 | C | 4 |
| algUup_Thalassiosira | 627 | 0.026 | 0.687 | 0.031 | 0.377 | M | 4 |
| algUup_Micromonas_sp | 531 | 0.093 | 0.243 | 0.083 | 0.594 | _ | 4 |
| algUup_Volvox_carter | 749 | 0.297 | 0.068 | 0.06 | 0.435 | _ | 5 |
| algUup_Micromonas_pu | 712 | 0.378 | 0.373 | 0.03 | 0.178 | C | 5 |
| algUup_Ostreococcus_ | 674 | 0.428 | 0.332 | 0.038 | 0.262 | C | 5 |
| algUup_Coccomyxa_sub | 551 | 0.087 | 0.226 | 0.021 | 0.826 | _ | 3 |
| algUup_Chondrus_cris | 758 | 0.756 | 0.295 | 0.006 | 0.066 | C | 3 |
| algUup_Emiliania_hux | 665 | 0.138 | 0.073 | 0.073 | 0.772 | _ | 2 |
| algUup_Aureococcus_a | 523 | 0.08 | 0.3 | 0.088 | 0.767 | _ | 3 |
| algEttA_Fragilariopsi | 555 | 0.066 | 0.12 | 0.109 | 0.845 | _ | 2 |
| algEttA_Fragilariopsi | 512 | 0.225 | 0.139 | 0.505 | 0.006 | S | 4 |
| algEttA_Pseudo-nitzsc | 546 | 0.08 | 0.076 | 0.236 | 0.822 | _ | 3 |
| algEttA_Ostreococcus_ | 549 | 0.018 | 0.116 | 0.417 | 0.75 | _ | 4 |
| algEttA_Chlamydomonas | 647 | 0.687 | 0.553 | 0.007 | 0.031 | C | 5 |
| algEttA_Phaeodactylum | 546 | 0.076 | 0.077 | 0.244 | 0.732 | _ | 3 |
| algEttA_Thalassiosira | 668 | 0.451 | 0.008 | 0.791 | 0.01 | S | 4 |
| algEttA_Micromonas_sp | 661 | 0.88 | 0.47 | 0.007 | 0.011 | C | 3 |
| algEttA_Volvox_carter | 750 | 0.514 | 0.407 | 0.001 | 0.031 | C | 5 |
| algEttA_Selaginella_m | 572 | 0.791 | 0.178 | 0.017 | 0.093 | C | 2 |
| algEttA_Selaginella_m | 541 | 0.025 | 0.25 | 0.261 | 0.664 | _ | 3 |
| algEttA_Micromonas_pu | 530 | 0.044 | 0.231 | 0.163 | 0.762 | _ | 3 |
| algEttA_Ostreococcus_ | 565 | 0.098 | 0.1 | 0.072 | 0.907 | _ | 1 |
| algEttA_Cyanidioschyz | 671 | 0.44 | 0.43 | 0.038 | 0.047 | C | 5 |
| algEttA_Coccomyxa_sub | 549 | 0.045 | 0.184 | 0.129 | 0.826 | _ | 2 |
| algEttA_Chondrus_cris | 620 | 0.702 | 0.217 | 0.016 | 0.038 | C | 3 |
| algEttA_Guillardia_th | 612 | 0.407 | 0.454 | 0.018 | 0.208 | M | 5 |
| algEttA_Aureococcus_a | 619 | 0.056 | 0.39 | 0.746 | 0.005 | S | 4 |
| cpYdif_Fragilariopsi | 613 | 0.215 | 0.078 | 0.1 | 0.707 | _ | 3 |
| cpYdif_Fragilariopsi | 613 | 0.215 | 0.078 | 0.1 | 0.707 | _ | 3 |
| cpYdif_Pseudo-nitzsc | 615 | 0.122 | 0.102 | 0.066 | 0.838 | _ | 2 |
| cpYdif_Oryza_sativa_ | 690 | 0.755 | 0.379 | 0 | 0.031 | C | 4 |
| cpYdif_Zea_mays_NP_0 | 705 | 0.876 | 0.271 | 0 | 0.02 | C | 2 |
| cpYdif_Arabidopsis_t | 678 | 0.336 | 0.115 | 0.014 | 0.013 | C | 4 |
| cpYdif_Arabidopsis_t | 692 | 0.695 | 0.156 | 0.004 | 0.179 | C | 3 |
| cpYdif_Ostreococcus_ | 649 | 0.139 | 0.086 | 0.046 | 0.911 | _ | 2 |
| cpYdif_Chlamydomonas | 592 | 0.283 | 0.083 | 0.062 | 0.782 | _ | 3 |
| cpYdif_Physcomitrell | 618 | 0.592 | 0.205 | 0.034 | 0.286 | C | 4 |
| cpYdif_Physcomitrell | 638 | 0.132 | 0.067 | 0.059 | 0.923 | _ | 2 |
| cpYdif_Phaeodactylum | 879 | 0.027 | 0.359 | 0.183 | 0.044 | M | 5 |
| cpYdif_Vitis_vinifer | 718 | 0.351 | 0.149 | 0.005 | 0.166 | C | 5 |
| cpYdif_Thalassiosira | 614 | 0.147 | 0.139 | 0.086 | 0.652 | _ | 3 |
| cpYdif_Populus_trich | 722 | 0.78 | 0.05 | 0.013 | 0.176 | C | 2 |
| cpYdif_Populus_trich | 700 | 0.863 | 0.079 | 0.006 | 0.234 | C | 2 |
| cpYdif_Sorghum_bicol | 703 | 0.789 | 0.382 | 0 | 0.017 | C | 3 |
| cpYdif_Micromonas_sp | 619 | 0.138 | 0.208 | 0.056 | 0.78 | _ | 3 |
| cpYdif_Ricinus_commu | 640 | 0.774 | 0.095 | 0.004 | 0.186 | C | 3 |
| cpYdif_Arabidopsis_l | 694 | 0.81 | 0.117 | 0.004 | 0.117 | C | 2 |
| cpYdif_Arabidopsis_l | 678 | 0.358 | 0.289 | 0.025 | 0.016 | C | 5 |
| cpYdif_Volvox_carter | 659 | 0.126 | 0.419 | 0.069 | 0.182 | M | 4 |
| cpYdif_Selaginella_m | 613 | 0.412 | 0.119 | 0.07 | 0.645 | _ | 4 |
| cpYdif_Selaginella_m | 613 | 0.412 | 0.119 | 0.07 | 0.645 | _ | 4 |
| cpYdif_Micromonas_pu | 619 | 0.091 | 0.238 | 0.065 | 0.809 | _ | 3 |
| cpYdif_Ostreococcus_ | 903 | 0.106 | 0.786 | 0.011 | 0.261 | M | 3 |
| cpYdif_Glycine_max_X | 696 | 0.466 | 0.165 | 0.017 | 0.231 | C | 4 |
| cpYdif_Glycine_max_X | 691 | 0.255 | 0.265 | 0.011 | 0.201 | M | 5 |
| cpYdif_Brachypodium_ | 690 | 0.565 | 0.302 | 0.004 | 0.052 | C | 4 |
| cpYdif_Medicago_trun | 700 | 0.634 | 0.126 | 0.019 | 0.099 | C | 3 |
| cpYdif_Cucumis_sativ | 714 | 0.498 | 0.191 | 0.005 | 0.106 | C | 4 |
| cpYdif_Solanum_lycop | 695 | 0.153 | 0.155 | 0.01 | 0.491 | _ | 4 |
| cpYdif_Fragaria_vesc | 705 | 0.751 | 0.31 | 0.001 | 0.033 | C | 3 |
| cpYdif_Cicer_arietin | 719 | 0.167 | 0.381 | 0.029 | 0.227 | M | 5 |
| cpYdif_Setaria_itali | 700 | 0.837 | 0.154 | 0.001 | 0.023 | C | 2 |
| cpYdif_Cyanidioschyz | 922 | 0.085 | 0.033 | 0.097 | 0.587 | _ | 3 |
| cpYdif_Coccomyxa_sub | 742 | 0.972 | 0.056 | 0.008 | 0.018 | C | 1 |
| cpYdif_Galdieria_sul | 798 | 0.316 | 0.191 | 0.023 | 0.386 | _ | 5 |
| cpYdif_Chondrus_cris | 960 | 0.746 | 0.511 | 0.02 | 0.013 | C | 4 |
| cpYdif_Emiliania_hux | 986 | 0.104 | 0.347 | 0.162 | 0.017 | M | 5 |
| cpYdif_Guillardia_th | 590 | 0.105 | 0.093 | 0.108 | 0.823 | _ | 2 |
| cpYdif_Chlorella_var | 605 | 0.132 | 0.039 | 0.025 | 0.852 | _ | 2 |
| cpYdif_Capsella_rube | 694 | 0.718 | 0.221 | 0.007 | 0.112 | C | 3 |
| cpYdif_Capsella_rube | 681 | 0.725 | 0.239 | 0.006 | 0.033 | C | 3 |
| cpYdif_Solanum_tuber | 695 | 0.132 | 0.127 | 0.018 | 0.418 | _ | 4 |
| cpYdif_Eutrema_salsu | 694 | 0.79 | 0.159 | 0.007 | 0.115 | C | 2 |
| cpYdif_Eutrema_salsu | 684 | 0.882 | 0.083 | 0.008 | 0.05 | C | 2 |
| cpYdif_Citrus_clemen | 692 | 0.878 | 0.125 | 0.004 | 0.062 | C | 2 |
| cpYdif_Citrus_sinens | 692 | 0.878 | 0.125 | 0.004 | 0.062 | C | 2 |
| cpYdif_Oryza_brachya | 660 | 0.651 | 0.129 | 0.007 | 0.362 | C | 4 |
| cpYdif_Amborella_tri | 710 | 0.422 | 0.062 | 0.01 | 0.496 | _ | 5 |
| cpYdif_Theobroma_cac | 690 | 0.665 | 0.576 | 0.002 | 0.034 | C | 5 |
| cpYdif_Theobroma_cac | 564 | 0.665 | 0.576 | 0.002 | 0.034 | C | 5 |
| cpYdif_Theobroma_cac | 486 | 0.7 | 0.369 | 0.009 | 0.046 | C | 4 |
| cpYdif_Phaseolus_vul | 701 | 0.624 | 0.149 | 0.011 | 0.166 | C | 3 |
| cpYdif_Prunus_persic | 716 | 0.364 | 0.356 | 0.005 | 0.166 | C | 5 |
| cpYdif_Prunus_mume_X | 657 | 0.37 | 0.357 | 0.006 | 0.16 | C | 5 |
| cpYdif_Malus_x_domes | 762 | 0.004 | 0.098 | 0.226 | 0.074 | S | 5 |
| cpYdif_Malus_x_domes | 393 | 0.076 | 0.26 | 0.09 | 0.769 | _ | 3 |
| cpYdif_Cucumis_melo_ | 714 | 0.449 | 0.162 | 0.011 | 0.132 | C | 4 |
| cpYdif_Phoenix_dacty | 712 | 0.322 | 0.354 | 0.001 | 0.325 | M | 5 |
| cpYdif_Aureococcus_a | 670 | 0.112 | 0.08 | 0.097 | 0.91 | _ | 2 |
| cpYdif_Brassica_rapa | 674 | 0.854 | 0.181 | 0.01 | 0.066 | C | 2 |
| cpYdif_Brassica_rapa | 687 | 0.695 | 0.316 | 0.002 | 0.072 | C | 4 |
| cpYdif_Pyrus_x_brets | 762 | 0.002 | 0.348 | 0.131 | 0.032 | M | 4 |
| cpYdif_Musa_acuminat | 712 | 0.458 | 0.22 | 0.004 | 0.245 | C | 4 |
| cpYdif_Musa_acuminat | 713 | 0.429 | 0.172 | 0.004 | 0.276 | C | 5 |
| cpYdif_Musa_acuminat | 712 | 0.429 | 0.172 | 0.004 | 0.276 | C | 5 |
| cpYdif_Nicotiana_tom | 693 | 0.739 | 0.129 | 0.011 | 0.09 | C | 2 |
| cpYdif_Nicotiana_syl | 693 | 0.609 | 0.193 | 0.007 | 0.097 | C | 3 |
| cpYdif_Eucalyptus_gr | 703 | 0.392 | 0.26 | 0.009 | 0.096 | C | 5 |
| cpYdif_Morus_notabil | 715 | 0.172 | 0.433 | 0.001 | 0.164 | M | 4 |
| cpYdif_Nelumbo_nucif | 711 | 0.403 | 0.095 | 0.016 | 0.107 | C | 4 |
| cpYdif_Nelumbo_nucif | 709 | 0.563 | 0.088 | 0.028 | 0.13 | C | 3 |
| cpYdif_Camelina_sati | 686 | 0.586 | 0.317 | 0.007 | 0.043 | C | 4 |
| cpYdif_Camelina_sati | 685 | 0.586 | 0.317 | 0.007 | 0.043 | C | 4 |
| cpYdif_Camelina_sati | 682 | 0.586 | 0.317 | 0.007 | 0.043 | C | 4 |
| cpYdif_Camelina_sati | 681 | 0.586 | 0.317 | 0.007 | 0.043 | C | 4 |
| cpYdif_Camelina_sati | 697 | 0.829 | 0.331 | 0.003 | 0.027 | C | 3 |
| cpYdif_Camelina_sati | 681 | 0.538 | 0.527 | 0.007 | 0.016 | C | 5 |
| cpYdif_Camelina_sati | 680 | 0.538 | 0.527 | 0.007 | 0.016 | C | 5 |
| cpYdif_Camelina_sati | 679 | 0.823 | 0.319 | 0.004 | 0.026 | C | 3 |
| cpYdif_Camelina_sati | 692 | 0.732 | 0.123 | 0.009 | 0.165 | C | 3 |
| cpYdif_Camelina_sati | 692 | 0.732 | 0.123 | 0.009 | 0.165 | C | 3 |
| cpYdif_Camelina_sati | 681 | 0.646 | 0.223 | 0.007 | 0.041 | C | 3 |
| mEttA_Phytophthora_c | 589 | 0.099 | 0.865 | 0.065 | 0.011 | M | 2 |
| mEttA_Phytophthora_c | 594 | 0.147 | 0.936 | 0.01 | 0.011 | M | 2 |
| mEttA_Phytophthora_i | 590 | 0.099 | 0.895 | 0.046 | 0.018 | M | 2 |
| mEttA_Emiliania_huxl | 676 | 0.221 | 0.148 | 0.026 | 0.832 | _ | 2 |
| mEttA_Saprolegnia_di | 564 | 0.029 | 0.907 | 0.014 | 0.131 | M | 2 |
| mEttA_Aphanomyces_in | 569 | 0.052 | 0.961 | 0.007 | 0.046 | M | 1 |
| mEttA_Phytophthora_p | 603 | 0.732 | 0.426 | 0.037 | 0.009 | C | 4 |
| mEttA_Phytophthora_s | 593 | 0.157 | 0.916 | 0.016 | 0.019 | M | 2 |
| mEttA_Aphanomyces_as | 586 | 0.053 | 0.921 | 0.012 | 0.106 | M | 1 |
| mEttA_Aphanomyces_as | 570 | 0.053 | 0.921 | 0.012 | 0.106 | M | 1 |

| Name | Sequence name truncated to 20 characters |
| --- | --- |
| Len | Sequence length |
| cTP, mTP, SP, other | Final NN scores on which the final prediction is based (Loc, see below). Note that the scores are not really probabilities, and they do not necessarily add to one. However, the location with the highest score is the most likely according to TargetP, and the relationship between the scores (the reliability class, see below) may be an indication of how certain the prediction is. |
| Loc | Prediction of localization, based on the scores above; the possible values are: |
| C | Chloroplast, i.e. the sequence contains cTP, a chloroplast transit peptide; |
| M | Mitochondrion, i.e. the sequence contains mTP, a mitochondrial targeting peptide; |
| S | Secretory pathway, i.e. the sequence contains SP, a signal peptide; |
| _ | Any other location; |
|  |  |
| Reliability class, from 1 to 5, where 1 indicates the strongest prediction. RC is a measure of the size of the difference ('diff') between the highest (winning) and the second highest output scores. There are 5 reliability classes, defined as follows: | |
| 1 : diff > 0.800 | |
| 2 : 0.800 > diff > 0.600 | |
| 3 : 0.600 > diff > 0.400 | |
| 4 : 0.400 > diff > 0.200 | |
| 5 : 0.200 > diff | |
| Thus, the lower the value of RC the safer the prediction. | |
